# A robust human norovirus replication model in zebrafish larvae

**DOI:** 10.1371/journal.ppat.1008009

**Published:** 2019-09-19

**Authors:** Jana Van Dycke, Annelii Ny, Nádia Conceição-Neto, Jan Maes, Myra Hosmillo, Arno Cuvry, Ian Goodfellow, Tatiane C. Nogueira, Erik Verbeken, Jelle Matthijnssens, Peter de Witte, Johan Neyts, Joana Rocha-Pereira

**Affiliations:** 1 KU Leuven–Department of Microbiology, Immunology and Transplantation, Rega Institute, Laboratory of Virology and Chemotherapy, Leuven, Belgium; 2 KU Leuven–Department of Pharmaceutical and Pharmacological Sciences, Laboratory for Molecular Biodiscovery, Leuven, Belgium; 3 KU Leuven–Department of Microbiology, Immunology and Transplantation, Rega Institute, Laboratory of Clinical and Epidemiological Virology, Leuven, Belgium; 4 University of Cambridge–Department of Pathology, Division of Virology, Addenbrooke's Hospital, Cambridge, United Kingdom; 5 KU Leuven–Department of Imaging & Pathology, Translational Cell & Tissue Research, Leuven, Belgium; 6 Global Virus Network (GVN), Centers of Excellence; Emory University, UNITED STATES

## Abstract

Human noroviruses (HuNoVs) are the most common cause of foodborne illness, with a societal cost of $60 billion and 219,000 deaths/year. The lack of robust small animal models has significantly hindered the understanding of norovirus biology and the development of effective therapeutics. Here we report that HuNoV GI and GII replicate to high titers in zebrafish (*Danio rerio*) larvae; replication peaks at day 2 post infection and is detectable for at least 6 days. The virus (HuNoV GII.4) could be passaged from larva to larva two consecutive times. HuNoV is detected in cells of the hematopoietic lineage and the intestine, supporting the notion of a dual tropism. Antiviral treatment reduces HuNoV replication by >2 log_10_, showing that this model is suited for antiviral studies. Zebrafish larvae constitute a simple and robust replication model that will largely facilitate studies of HuNoV biology and the development of antiviral strategies.

## Author summary

Human norovirus (HuNoV) is the number one agent of viral gastroenteritis worldwide. It can infect people of all age groups, resulting in 700 million infections and 219,000 deaths each year. Outbreaks of acute HuNoV gastroenteritis occur often, but chronic infections also happen in people with immune deficiencies. Despite its clinical relevance, studying the virus has been a decades-long challenge because no simple and efficient cultivation system existed. This has recently started to change; we here contribute with an important step forward by establishing an *in vivo* model system to study HuNoV replication using zebrafish larvae. We inject a HuNoV in the yolk (food reserve) of 3-day-old zebrafish larvae, which results in high virus titers up to 6 days after inoculation. We could detect the virus in the tissues of the infected larvae by a variety of techniques including histology, which showed us in which organs the virus is present. Importantly, by adding an antiviral to the water in which the larvae swim, we could significantly reduce the virus levels in the larvae. This means that testing small molecules and developing the first antiviral therapy for HuNoV will be much easier from hereon.

## Introduction

Human noroviruses (HuNoVs) are an important cause of epidemic and sporadic acute gastroenteritis worldwide; annually about 700 million people develop a HuNoV infection resulting in ~219,000 deaths and a societal cost estimated at 60 billion US dollars [1]. Large outbreaks of norovirus gastroenteritis are frequent and have a significant impact in terms of morbidity, mortality and health care costs, in particular in hospital wards and nursing homes. Chronic norovirus infections present a problem for a large group of immunodeficient patients, who may present with diarrhea for several months. Furthermore, in countries where routine rotavirus vaccination has been implemented, noroviruses are the most common cause of severe childhood diarrhea resulting in important morbidity and mortality [2]. Knowledge on the biology and pathogenesis of human noroviruses largely depends upon the development of robust and physiologically relevant cultivation systems. The available model systems carry important limitations. HuNoV replication has been reported in large animals such as chimpanzees, gnotobiotic pigs and calves. However, these animals are either not suited for extensive studies or are, in the case of chimpanzees no longer allowed due to ethical reasons [3–5]. Importantly, a HuNoV mouse model was described in BALB/c Rag-γ c-deficient mice, but only a short-lasting replication was achieved, which limits its applications [6]. Standard cell culture models are to date not available, but first steps towards this have been given by establishing that (i) human B-cells are susceptible to HuNoV and that (ii) HuNoV can be cultivated in stem-cell-derived enteroids [7–9]. There is thus an urgent need for simpler, more robust, widely available HuNoV replication models. Such models should contribute to a better understanding of the biology of HuNoV replication and infection, this will significantly facilitate larger-scale research efforts, such as the development of therapeutic strategies.

Zebrafish (*Danio rerio*) are optically-transparent tropical freshwater fish of the family *Cyprinidae* that are widely used as vertebrate models of disease. They have remarkable genetic, physiologic and pharmacologic similarities to humans. Compared to rodents, the maintenance and husbandry costs are very low. Zebrafish have high fecundity and using their offspring is in better compliance with the 3Rs principles of humane animal experimentation (EU Directive 2010/63/EU). The immune system of zebrafish is comparable to that of humans; there are B and T cells, macrophages, neutrophils and a comparable set of signaling molecules and pathways [10]. Whereas innate immunity is present at all developmental stages, adaptive immunity develops after 4–6 weeks of life [11, 12]. Host-pathogen interactions can be studied, as zebrafish are naturally infected by multiple bacteria, protozoa and viruses that affect mammals [11]. Infection of zebrafish larvae has been shown with some human viruses (herpes simplex virus type 1, influenza A virus, and chikungunya virus) [13–15] as well as enteric bacteria, e.g. *E*. *coli*, *Listeria*, *Salmonella*, *Shigella* and *Vibrio* [11]. The intestinal tract of zebrafish is comprised of large folds of an epithelial lining, a *lamina propria* containing immune cells and underlying smooth muscle layers [16]. Enterocytes, goblet cells, enteroendocrine cells, and possibly M-cells are present, but not Paneth cells or Peyer’s patches [12]. Intestinal tuft cells, which were recently shown to be a target cell for the mouse norovirus (MNV) [17], have been described in teleost fish thus are likely present in zebrafish. There is an intestinal bulb (instead of a stomach) and a mid- and posterior intestine [16]. Epithelial cells show a high-turnover from base to tip, with intestinal epithelial stem cells at the base and apoptotic cells at the tips [16]. A resident commensal microbiota is present (comprising most bacterial phyla of mammals) and serve analogous functions in the digestive tract [11, 16]. Here we report a robust replication model of HuNoV in zebrafish larvae.

## Results

### A HuNoV GII.P7-GII.6 replicates in zebrafish larvae

**Fig 1:**
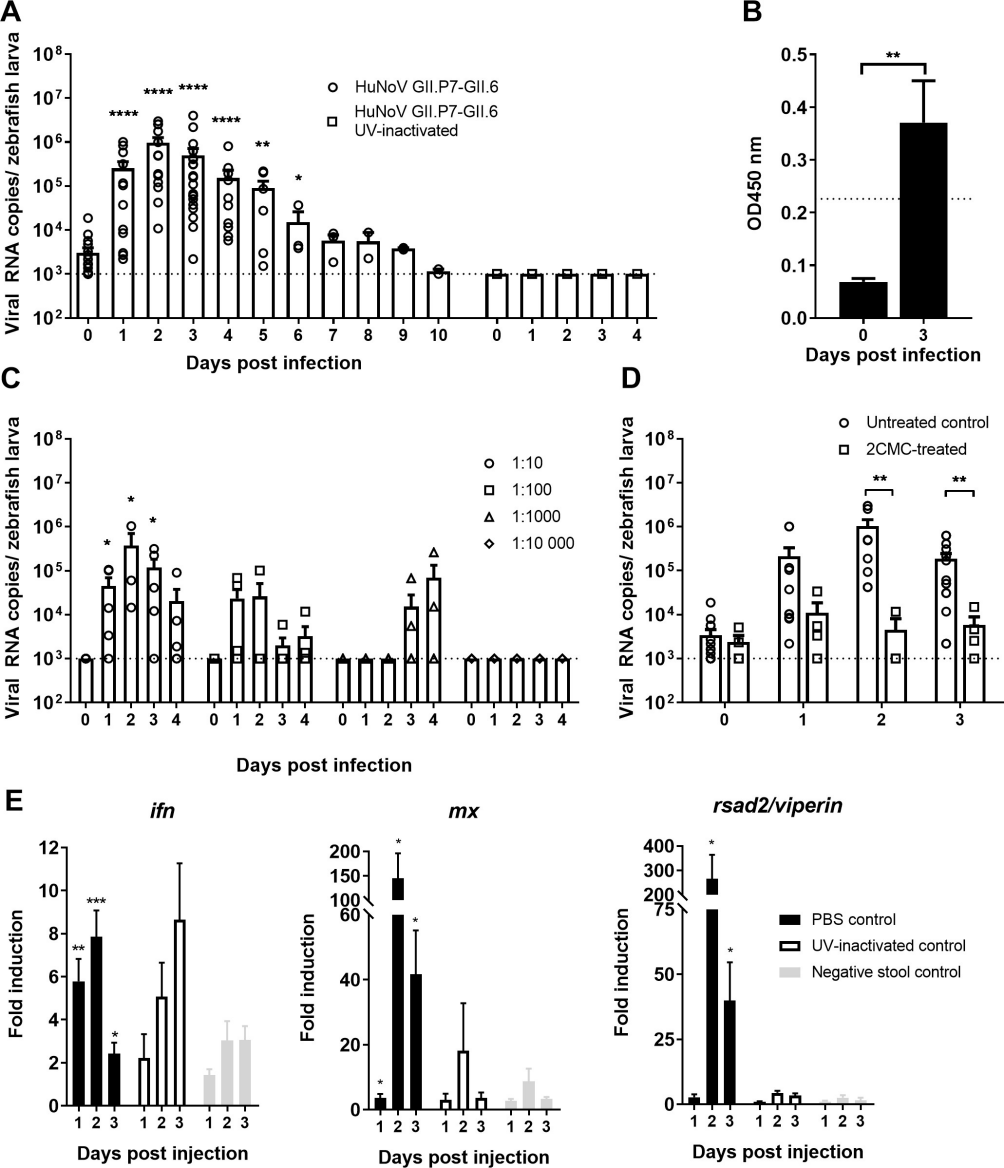
HuNoV GII.P7-GII.6 replicates in zebrafish larvae. (**A**) Injection of zebrafish larvae with HuNoV GII.P7-GII.6 (22 independent experiments) or UV-inactivated virus (2 independent experiments). Bars represent viral RNA levels/zebrafish larva, quantified by RT-qPCR. The dotted line represents the limit of detection (LOD). Viral RNA levels in larvae injected with the UV-inactivated sample was set at the LOD (undetected by RT-qPCR). (**B**) Viral antigens quantified by ELISA in HuNoV GII.P7-GII.6-injected larvae. Bars represent OD values/10 larvae. The dotted line is the calculated cutoff+10%, above which samples are considered positive. (**C**) Zebrafish larvae injected with serial dilutions of HuNoV GII.P7-GII.6 (5 independent experiments). Bars represent viral RNA levels/zebrafish larva, quantified by RT-qPCR. The dotted line represents the LOD. (**D**) HuNoV GII.P7-GII.6-injected larvae treated with 4 mM of 2’*-C*-methylcytidine via immersion in Danieau’s solution (4 independent experiments). Bars represent the viral RNA levels/zebrafish larva, quantified by RT-qPCR. (**E**) The effect of HuNoV GII.P7-GII.6 replication on the expression of *ifn*, *mx*, and *rsad2/viperin*, determined by RT-qPCR. Bars represent the fold-induction in HuNoV-injected larvae, compared to zebrafish larvae injected with PBS (black bars), UV-inactivated HuNoV GII.P7-GII.6 (empty bars) or a stool sample negative for the presence of HuNoV (grey bars) and normalized to the housekeeping genes (4–9 independent experiments). For all graphs: in every independent experiment 10 zebrafish larvae were harvested at each time point, mean values ± SEM are presented, Mann-Whitney test, where ****p<0.0001, ***p<0.001, **p<0.01, *p<0.05.

Zebrafish larvae were injected with a PBS suspension of a HuNoV positive stool sample at 3 days post-fertilization (dpf). At this time point, zebrafish larvae have hatched and organs are formed (including the full-length gastrointestinal tract). Three nL, containing 3.4 x 10^6^ viral RNA copies of HuNoV GII.P7-GII.6 (1.1 x 10^13^ RNA copies/g of stool), were injected in the yolk of the larvae (which provides nutrition during early larval stage). Each day post-infection (pi), the general condition of the zebrafish larvae was assessed microscopically and these were harvested in groups of 10 for viral RNA quantification by RT-qPCR [14, 15]. To detect input virus, in every independent experiment, 10 larvae were harvested at day 0 pi (specifically 1 h pi). A maximum increase of ~2.5 log_10_ in viral RNA copies compared to day 0 was detected at day 2 pi (Fig 1A); high levels of viral RNA remained detectable for at least 6 days pi (Fig 1A). When larvae were injected with 3 nL of UV-inactivated HuNoV GII.P7-GII.6, no increase in viral RNA titers was detected (Fig 1A). No obvious signs of distress or disease were observed as a result of the viral replication (e.g. changes in posture, swimming behavior or signs of edema). To determine the 50% infectious dose (ID_50_) for this strain, larvae were injected with 10-fold dilution series of the virus (Fig 1C). The ID_50_ was calculated to be 1.8 x 10^3^ viral RNA copies. HuNoV antigens were detected using the commercial enzyme immunoassay (EIA) RIDASCREEN (R-Biopharm), in HuNoV GII.P7-GII.6-injected zebrafish larvae harvested at day 3 pi (Fig 1B). 

We next investigated the innate immune response to a HuNoV infection of larvae at multiple time points pi. An increased expression of *ifn*, *mx and rsad2/viperin* mRNA was detected, with a 8-fold, 144-fold and 266-fold maximum increase, respectively, when compared to PBS injected larvae (Fig 1E). Two additional controls were included, namely (i) larvae injected with a UV-inactivated virus-containing Stool sample and (ii) larvae injected with a stool sample that was negative for HuNoV (Fig 1E). When UV-inactivated virus was injected, a delayed increase of *ifn* was observed, but this did not trigger the downstream cascade partners *mx* and *rsad2* (Fig 1E). The level of upregulation reported here is in line with the observed induction of the *ifn* response following an influenza A infection of zebrafish larvae [14]. These same genes (or the related cytokines) were upregulated in other *in vivo* models, such as in HuNoV-infected calves or MNV-infected mice [5, 18], or in a HuNoV replicon system in the case of viperin [19]. Altogether, this points out that the antiviral signaling cascades that are activated upon a HuNoV infection of zebrafish larvae are relevant and likely the same as in humans. Zebrafish are thus a suitable model for the study of the innate immune response to a HuNoV infection.

Next, injected larvae were treated with a broad-spectrum antiviral, i.e. the viral polymerase inhibitor 2’-*C*-methylcytidine (2CMC) of which we showed earlier inhibition of MNV replication *in vitro* and in mice [20, 21], by immersion (whereby the molecule was added to the water). A 2.4 log_10_ reduction in viral RNA titers was observed at the peak of replication (Fig 1D). The fact that replication can be significantly reduced with an inhibitor of the viral polymerase provides further evidence that HuNoV replicates efficiently in zebrafish larvae and that the model is suitable for antiviral drug development.

### Viral metagenomics analysis of the HuNoV GII.P7-GII.6 stool samples

**Fig 2:**
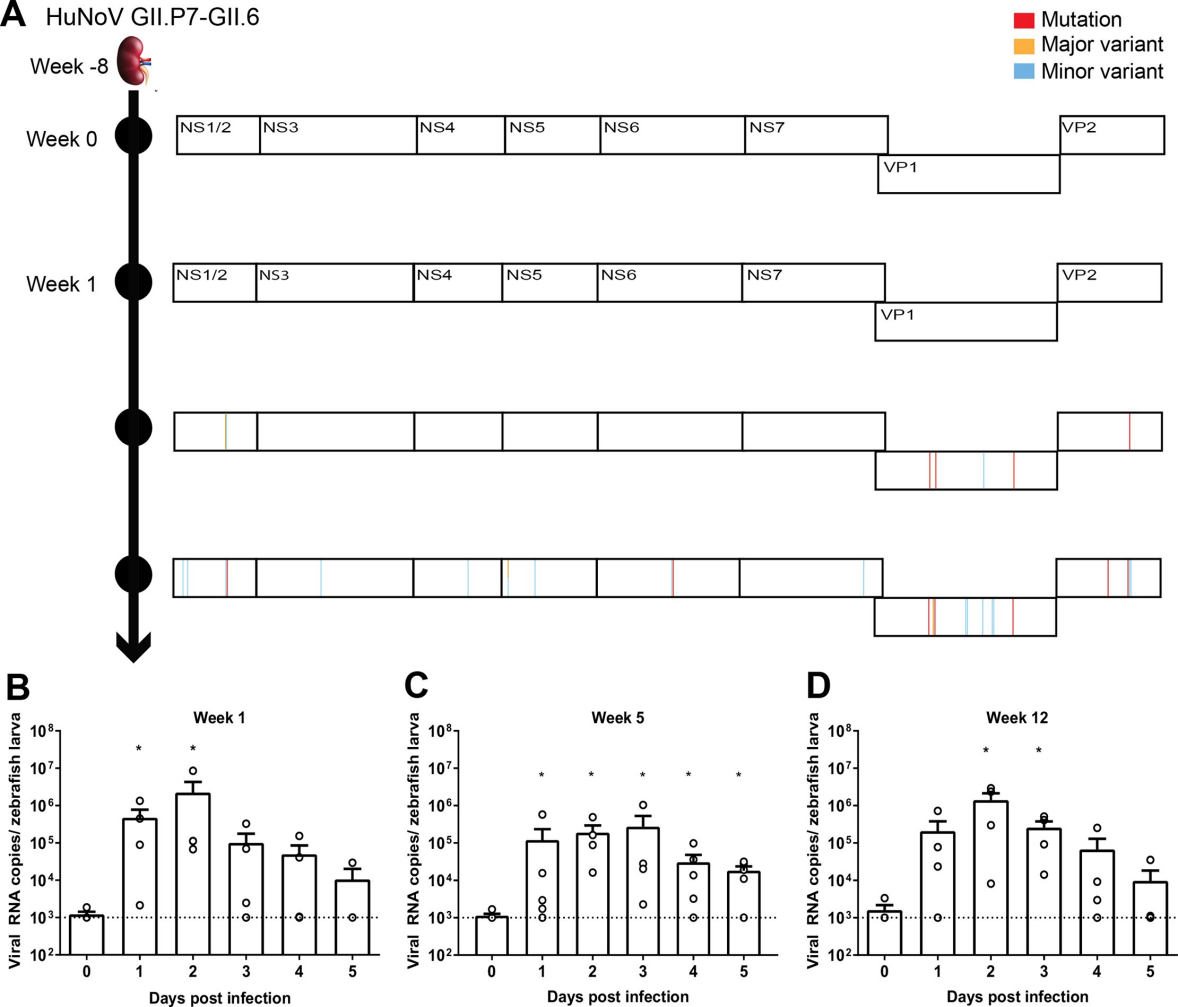
Characterization of the HuNoV GII.P7-GII.6 sample and clinical history of the patient. (**A**) A 2.5-year-old patient that had received a kidney transplant and presented 8 weeks later with acute viral diarrhea caused by HuNoV GII.P7-GII.6. The infection lasted for ~7 months along which multiple stool samples were collected; four of these samples were characterized (week 0, week 1, week 5 and week 12). The immunosuppressive therapy consisted of tacrolimus and mycophenolate. The complete HuNoV genomes were determined by deep sequencing using the NextSeq500 platform (Illumina) and whole genome sequence analysis was performed in comparison to week 0. Mutations, major and minor variants (respectively, ≥80%, 50–80% and 10–49% of the reads had a different nucleotide) are depicted for the HuNoV strains present in samples from week 1, 5 and 12 pi. (**B-D)** Zebrafish larvae injected with HuNoV GII.P7-GII.6 of week 1, 5 or 12 (4–5 independent experiments). The calculated inocula (3 nL per zebrafish larvae) were (**B**) 2.2 x 10^4^ (**C**) 2.5 x 10^3^ and (**D**) 4.4 x 10^4^ viral RNA copies. Bars represent viral RNA levels/zebrafish larva, quantified by RT-qPCR. The dotted line represents the LOD. For all graphs: in every independent experiment 10 zebrafish larvae were harvested at each time point, mean values ± SEM are presented, Mann-Whitney test, where *p<0.05.

One of the hurdles to develop robust replication models for HuNoV is the need to use a stool sample of an infected patient as inoculum. In order to rule out any potential impact of other agents present in the sample we fully characterized the samples used. A viral metagenomics analysis was performed on the clinical sample containing the HuNoV GII.P7-GII.6 used in this study, together with subsequent clinical samples of the same chronically-infected 2.5-year old transplant patient (Fig 2). The viral population consisted predominantly of HuNoV (with a minor presence of anelloviruses, common in patients undergoing immunosuppressive therapy [22]). Mutations that occurred in the virus over the course of the infection were mostly in the capsid-encoding region (Fig 2, S1 Table) and did not affect the kinetics of virus replication in larvae (Fig 2B–2D). In addition, upon injection of HuNoV GII.P7-GII.6 (week 0) in zebrafish larvae no mutations were detected in the viral genome at the peak of replication (2 days pi).

### Replication of additional genotypes of HuNoV GI and GII

**Fig 3:**
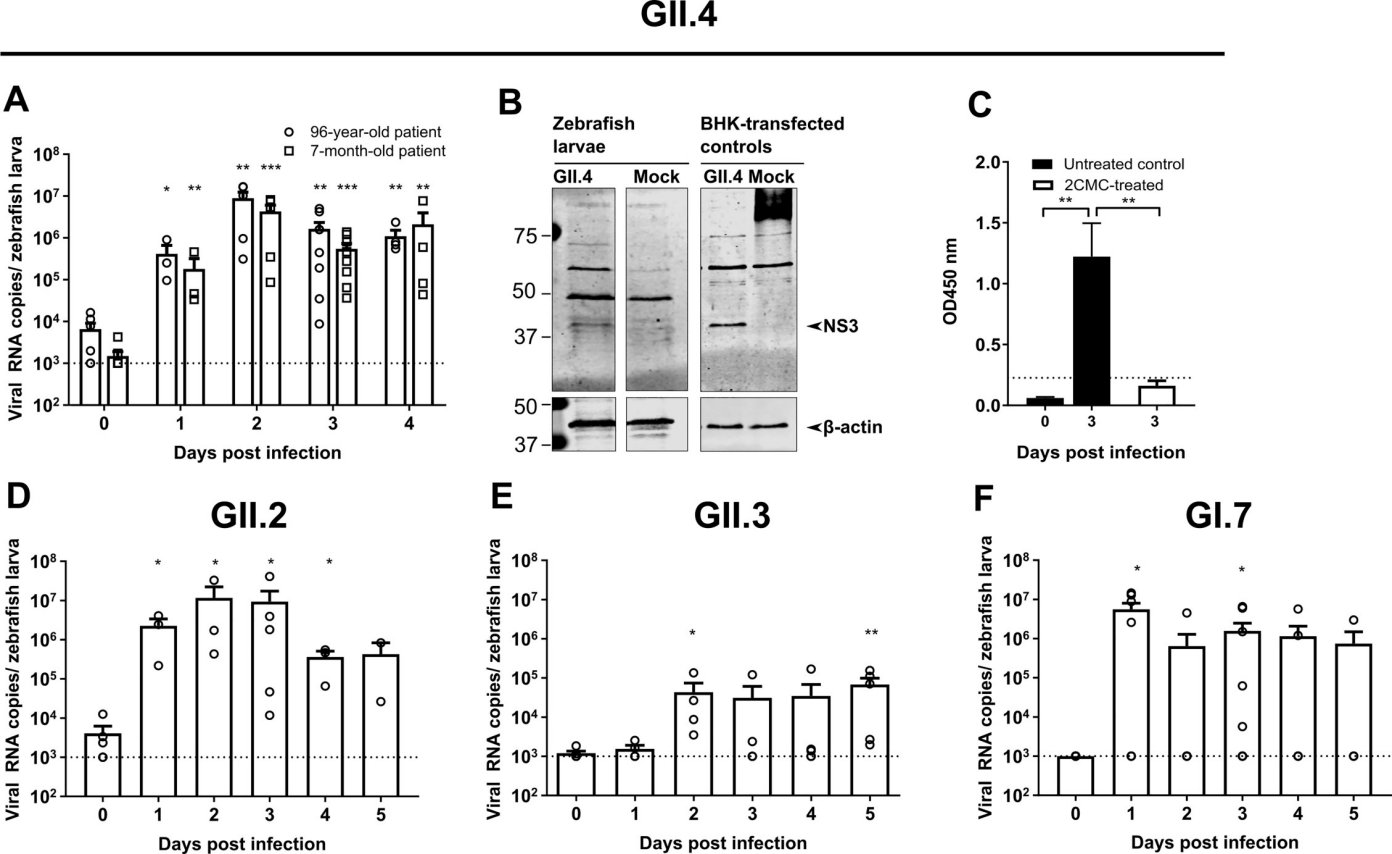
Injection of zebrafish larvae with HuNoV GI and GII of other genotypes. (**A**) Zebrafish larvae injected with GII.P4 New Orleans-GII.4 Sydney, from a 96-year-old patient and a 7-month-old patient [6 independent experiments]. Bars represent viral RNA levels/zebrafish larva, quantified by RT-qPCR. The dotted line represents the limit of detection (LOD). (**B**) The viral NS3 protein detected in HuNoV GII.4-injected larvae by western blot analysis, BHK cells transfected with a GII.4 construct were used as positive control. (**C**) Structural antigens were detected by EIA [here 2CMC-treated HuNoV GII.4-injected zebrafish were also included]. Bars represent OD values/10 larvae. The dotted line is the calculated cutoff +10%, above which samples are considered positive. (**D**) Zebrafish larvae injected with HuNoV GII.P16-GII.2 (from an 87-year-old patient) [5 independent experiments]. Bars represent the viral RNA levels/zebrafish larva, quantified by RT-qPCR. (**E**) Zebrafish larvae injected with GII.P16-GII.3 (from a 3.5-year-old patient) [5 independent experiments]. Bars represent the viral RNA levels/zebrafish larva, quantified by RT-qPCR. (**F**) Zebrafish larvae injected with HuNoV GI.P7-GI.7 (from a 52-year-old patient) [7 independent experiments]. Bars represent the viral RNA levels/zebrafish larva, quantified by RT-qPCR. The dotted line represents the LOD. In all graphs: in every independent experiment 10 larvae were harvested at each time point, mean values ± SEM are presented, Mann-Whitney test, where ***p<0.001, **p<0.01, *p<0.05.

Injection of zebrafish larvae with other HuNoV genotypes was next performed. Injection with the HuNoV GII.P4 New Orleans-GII.4 Sydney strain, recovered from stool samples of two different patients, resulted in a 3.1 and 3.4 log_10_ increase in viral replication in both cases. A maximum of ~10^7^ viral RNA copies/zebrafish larva was detected at day 2 pi (Fig 3A), the highest observed in this model. Viral non-structural and structural antigens were detected by western blot (Fig 3B, S1 Fig) and by EIA (Fig 3C), respectively. Viral antigens were no longer detected by EIA in 2CMC-treated HuNoV GII.4-infected larvae (Fig 3C). Injection with HuNoV GII.P16-GII.2 (Fig 3D) and GII.P16-GII.3 (Fig 3E) yielded increasing viral RNA titers, although the replication kinetics of GII.P16-GII.3 reached lower titers and at a later time point than that observed for the other genotypes. Slower kinetics of HuNoV GII.3 replication was also observed in stem-cell-derived enteroids [23]. A GI HuNoV, specifically GI.P7-GI.7, replicated with comparable kinetics to GII viruses (Fig 3F). Injection of zebrafish larvae with MNV (genogroup V) yielded no productive infection (S2 Fig), most likely due to the fact that the receptors CD300lf and CD300ld are not encoded by zebrafish [24].

**Fig 4:**
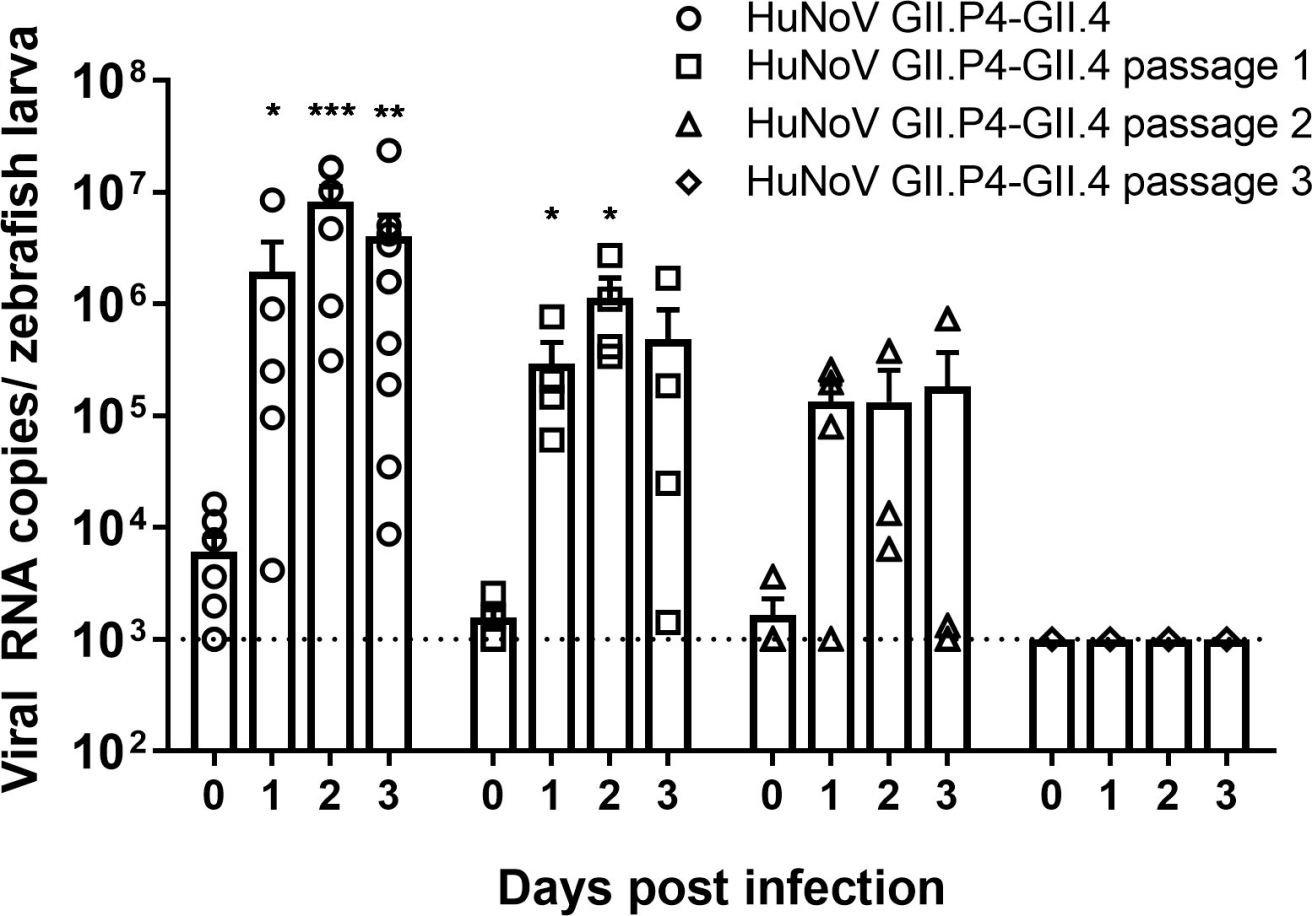
Serial passaging of HuNoV GII.P4-GII.4 in zebrafish larvae. Bars represent viral RNA levels/larva after serial passaging of HuNoV GII.P4 New Orleans-GII.4 Sydney, quantified by RT-qPCR [5 independent experiments]. The dotted line represents the limit of detection (LOD). In every independent experiment 10 larvae were harvested at each time point, mean values ± SEM are presented, Mann-Whitney test, where ***p<0.001, **p<0.01, *p<0.05.

We next passaged the virus from larva to larva. To that end HuNoV GII.P4-GII.4 injected zebrafish larvae was collected at the peak of replication, homogenized in 50 μL PBS, and the supernatant was used to inject new larvae. This was successful for two passages (Fig 4).

### Infection route and sites of replication of HuNoV in zebrafish larvae

**Fig 5:**
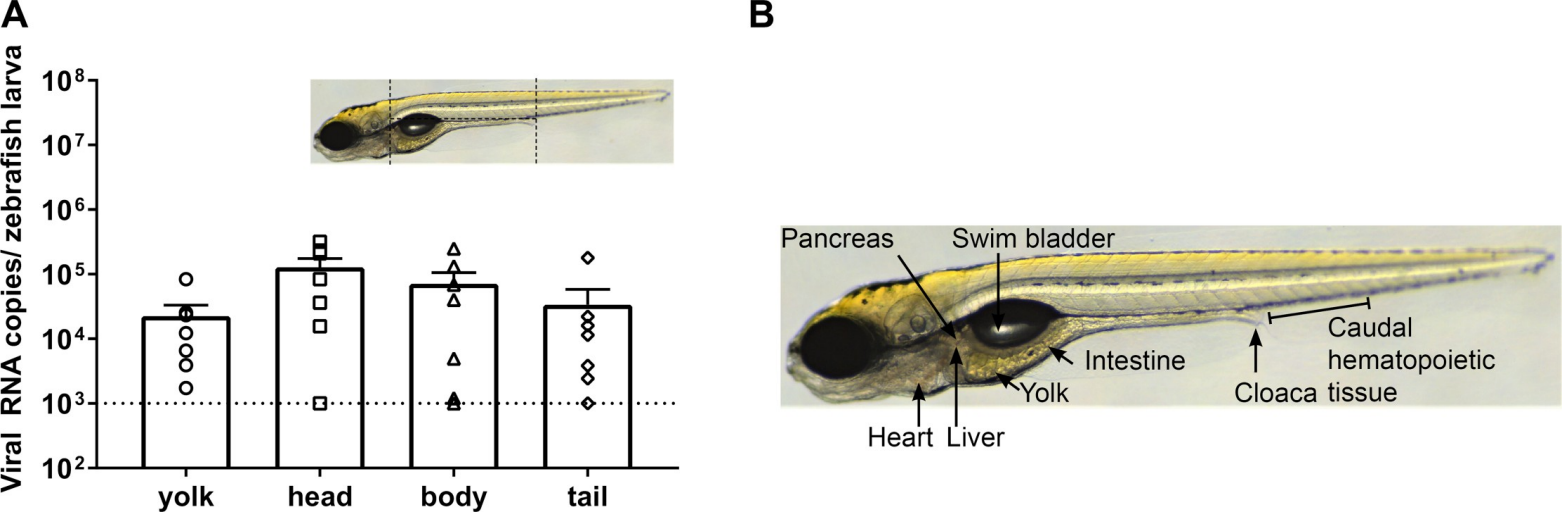
HuNoV sites of replication. (**A**) Ten HuNoV-injected larvae were deyolked and then dissected into head, body, and tail (as depicted in the scheme) at 3 days pi. Bars represent the viral RNA levels/zebrafish larva, quantified by RT-qPCR (7 independent experiments). (**B**) Anatomy of a 6 dpf zebrafish larva.

**Fig 6:**
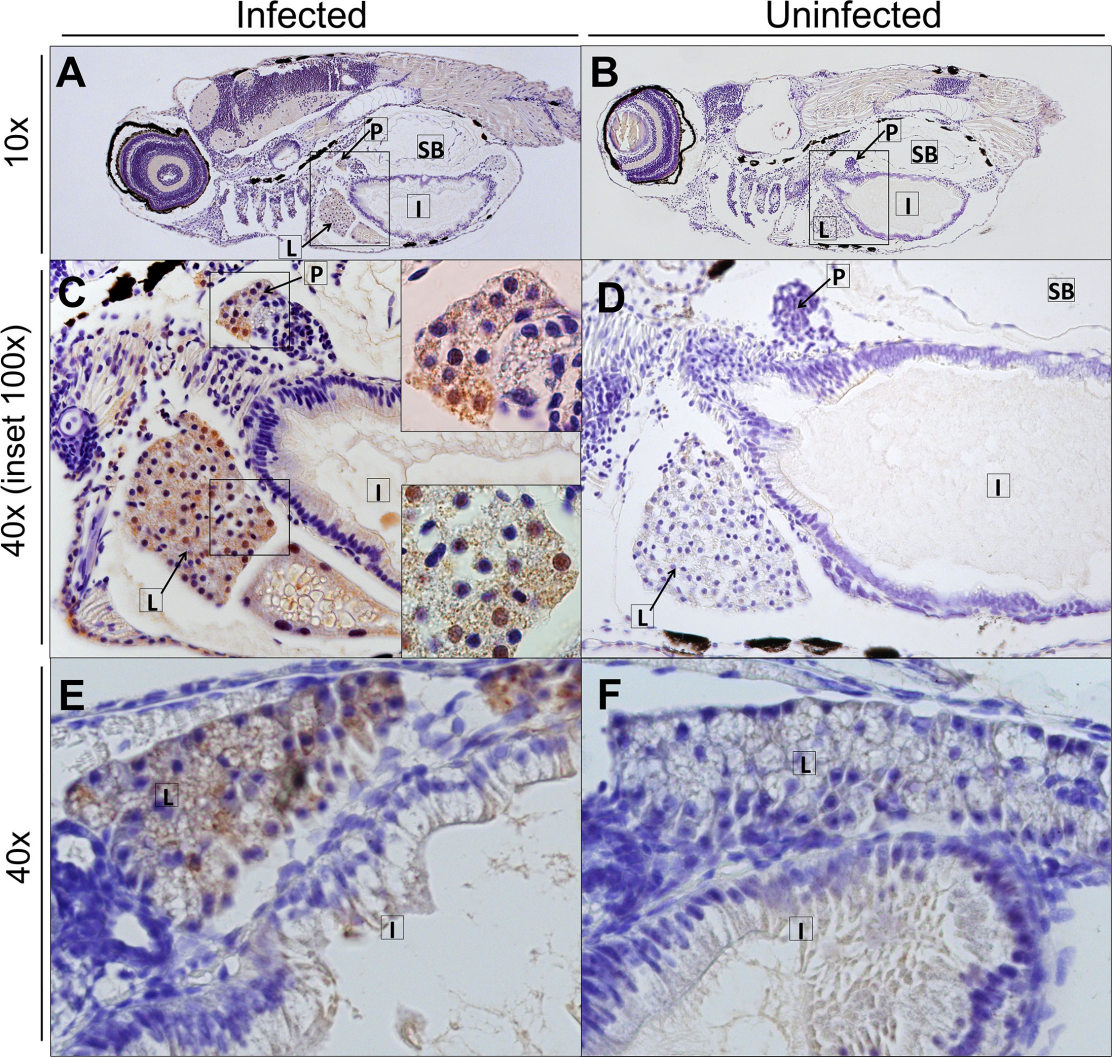
HuNoV detected in the liver and pancreas of infected zebrafish larvae at 3 days pi. Immunohistochemistry of HuNoV GII.P7-GII.6-infected zebrafish larvae (**A, C, E**) harvested at day 3 pi [and the respective uninfected controls **(B, D, F**)] was performed. Images show 5 μm sections stained with antibodies targeting VP1 at 10x and 40x magnifications, insets at 100x magnification of sagittal sections (**A-D**) and coronal sections (**E-F**). Viral antigens were detected in the liver (**A, C, D, E**) and pancreas (**A, C**). L: liver, P: pancreas, SB: swim bladder, I: intestine.

**Fig 7:**
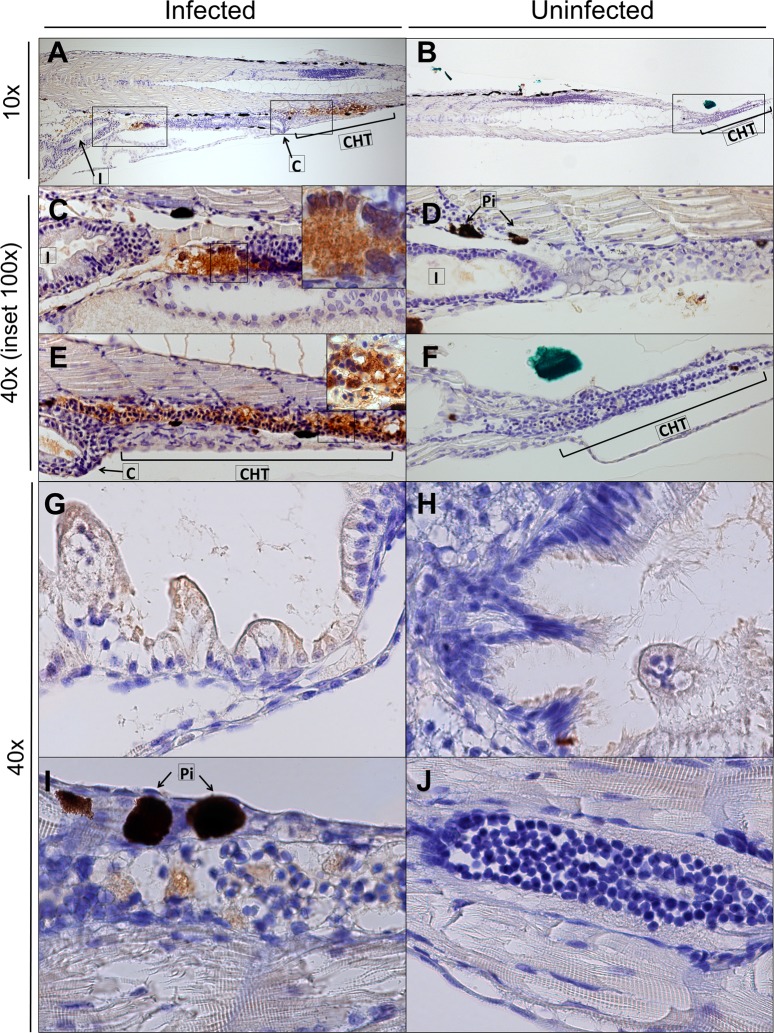
HuNoV detected in the intestine and caudal hematopoietic tissue of infected zebrafish larvae at 3 days pi. Immunohistochemistry of HuNoV GII.P7-GII.6-infected zebrafish larvae (**A, C, E, G, I**) harvested at day 3 pi [and the respective uninfected controls (**B, D, F, H, J**)] was performed. Images show 5 μm sections stained with antibodies targeting VP1 at 10x and 40x magnifications, insets at 100x magnification of sagittal sections (**A-F**) and coronal sections (**G-J**). Viral antigens were detected in the intestine (**A, C, G**) and caudal hematopoietic tissue (**A, E, I**). L: liver, C: cloaca, SB: swim bladder, I: intestine, Pi: pigment, CHT: caudal hematopoietic tissue.

Although a yolk injection adds a known virus inoculum to the food of the larvae and is therefore close to the natural route of infection, we next attempted to infect larvae by adding virus to the swimming water *(i*.*e*. *via* immersion). This was done by immersing 4 or 5 dpf larvae (when the mouth has opened and the gastrointestinal tract fully matured) in 1 mL of a HuNoV GII.P4-GII.4 PBS suspension for 8 h, after which the larvae were washed extensively with Danieau’s. No consistent increase in viral replication was noted up to day 5 pi. To determine the preferential site of replication of HuNoV in zebrafish larvae, HuNoV GII.P7-GII.6-injected larvae were dissected at day 3 pi in 4 different parts (yolk, head, body and tail). Viral RNA titers were detected in every part (Fig 5A), whereby the yolk (the initial site of inoculation) had the lowest titers, implying that HuNoV disseminates past the yolk and intestine. To investigate which tissues are infected, sagittal and coronal histological sections of larvae injected with HuNoV GII.P7-GII.6 were harvested at day 3 pi and stained with HuNoV VP1-specific antibodies (Figs 6 and 7, S3 Fig). Viral antigens were frequently detected in the intestine, pancreas and liver. A strong signal was observed in the caudal hematopoietic tissue (CHT), which contains hematopoietic stem/progenitor cells (HSPCs) that differentiate into multiple blood lineages and by 4 dpf start to migrate to the kidney marrow and thymus [25]. This migration may explain why HuNoV was detected in every section of the larvae. HuNoV has been detected in the intestine and liver of chimpanzees [3], and has as well been reported to replicate in cells of hematopoietic lineage [26]. To determine if viral antigens could be detected at earlier time points, staining’s of larvae were performed at day 1 and 2 pi. At 1 day pi, the intestine (anterior and posterior) was already strongly positive (S4 Fig). Lighter staining was detected in the liver (S4A Fig), pancreas (S4A Fig) and caudal hematopoietic tissue (CHT) (S4G Fig). The same was observed at day 2 pi (S4C and S4E Fig).

## Discussion

Here we describe a robust and very convenient HuNoV replication model in zebrafish larvae. Zebrafish share about ~70% of their genes with humans and 82% of disease-related genes have at least one zebrafish orthologue [27], but there are obvious differences between zebrafish larvae and the natural host. Zebrafish larva are an optically accessible whole-organism with comparable organs and systems as higher vertebrates, thus providing a unique system to identify and study physiologically relevant features of a HuNoV infection. Zebrafish-based models have also been used to study multiple pathogenic bacteria. Such studies have contributed to a better understanding of cellular microbiology, for example by highlighting the occurrence of emergency granulopoiesis for the replenishment of neutrophils after a *Salmonella* infection [28] and by *in vivo* imaging of septin cage entrapment of *Shigella flexneri* leading to autophagy [29].

We here report that HuNoV of multiple genotypes replicate efficiently in zebrafish larvae, with GII.4 viruses yielding the highest fold-increase over background (˃ 3 log_10_). This is much higher than the 60-fold increase observed following intraperitoneal injection of HuNoV in BALB/c Rag-γ c mice [30]. Also when compared to the use of intestinal enteroids, where infection with HuNoV GII.4 yielded a 1.5–2.5 log_10_ increase of viral RNA [or a maximum average of 2.8 log_10_ after the addition of bile] [7], we here consistently detected a stronger increase of viral genomes. We here also report replication of HuNoV GII.6 in zebrafish larvae (this was not reported in intestinal enteroids [23, 31]). In addition, replication of GII.3 viruses was achieved without the need for addition of bile, although it should be noted that the yolk is a lipid-rich structure [32]. In addition, a HuNoV GI (genotype GI.7) replicated efficiently, yielding a comparable 3 log_10_ increase in viral RNA with kinetics comparable to that observed for GII viruses. Only HuNoV GI.1 was ever shown to replicate and this to a maximum yield of 1 log_10_ (with addition of bile) [23, 31]. This finding highlights a unique opportunity to study the biology of GI noroviruses.

In addition, we succeeded to passage HuNoV GII.4 from larva to larva, which was possible up to the second passage. The reason this was not possible for more passages is that a minimum volume of PBS (50 μL) is needed to homogenize the zebrafish tissue whereas only 3 nL can be injected in each larva. This dilution after each homogenization (~16,600-fold) results in a too low inoculum in the third passage (below the ~10^3^ viral RNA copies determined as the ID_50_ for a GII.6 strain). This is thus the main limiting factor, although other unknown restriction factors could exist.

HuNoV replication was most prominent in the intestine of larvae one day pi, as detected by immunohistochemistry. Viral antigens were also detected in the hematopoietic tissue at this point and this evolved to very intense staining at day 3 pi. These findings clearly illustrate virus replication in both tissues thus supporting a notion of dual tropism of HuNoV. The fact that there are common features to the innate immune response triggered by the virus infection in zebrafish further adds value to the model. In addition, their optical transparency allows live imaging studies, for example using transgenic lines with tagged immune or gut cells, which would aid studies of tissue tropism and pathogenesis. The availability of simple genetic manipulation methods facilitates the understanding of gene functions [33, 34], which, combined with the availability of many knockout alleles [35], could significantly enhance our ability to dissect HuNoV-host interactions.

Zebrafish are widely available at universities/research centers and their use is amenable to high-throughput studies. A trained researcher can inject/manipulate hundreds of larvae per day requiring only a microscope, a micromanipulator and injection pump. Moreover, only a few nL of virus is required to inject zebrafish larvae. Consequently, a 100 mg stool aliquot (with a high virus titer) is sufficient to inject about 300,000 larvae, yielding 30,000+ data points. The ability to generate large and homogenous datasets is essential for large-scale efforts such as the development of therapeutics [36–38]. The zebrafish larvae fit in 96- and 384-well plates and small molecules can be simply added to the swimming water. Moreover, only a minute amount of compound is needed to assess a potential antiviral activity, this is in stark contrast to what is needed for studies in mice. Thus the model here validated for antiviral drug studies using GI and GII HuNoVs, now allows to readily assess the potential antiviral activity of novel inhibitors.

Overall, this model is a major step forward in the study of HuNoV replication and provides the first robust small laboratory animal of HuNoV infection.

## Materials and methods

### Ethics statement

All zebrafish experiments were approved and performed according to the rules and regulations of the Ethical Committee of KU Leuven (P086/2017), in compliance with the regulations of the European Union (EU) concerning the welfare of laboratory animals as declared in Directive 2010/63/EU. Zebrafish larvae were used from 48 hpf until a maximum of 144 hpf. Human stool samples, positive for human norovirus (HuNoV), were obtained from the existing collection of samples of the University Hospital of Leuven (Belgium) in an anonymous way. In a letter of consent, the patient is informed that leftover stool samples can be used for scientific research; no new samples were collected in light of this study.

### Zebrafish maintenance

Wild type AB adult zebrafish were maintained in the aquatic facility of the KU Leuven (temperature of 28 °C and 14/10 h light/dark cycle). Fertilized eggs were collected from adults placed in mating cages and kept in petri dishes containing Danieau’s solution (1.5 mM HEPES, 17.4 mM NaCl, 0.21 mM KCl, 0.12 mM MgSO_4_, and 0.18 mM Ca(NO_3_)_2_ and 0.6μM methylene blue) at 28 °C until the start of experiments.

### Collection and processing of HuNoV stool samples

Human stool samples, positive for human norovirus (HuNoV), were obtained from the University Hospital of Leuven (Belgium). An aliquot of 100 mg of each stool sample was re-suspended in 1 mL of sterile PBS, thoroughly vortexed and centrifuged (5 min, 1,000 g), supernatant was harvested and stored at -80°C. This virus suspension was used for RNA extractions, quantification by RT-qPCR, sequencing and injections in the zebrafish larvae. HuNoV RNA was extracted from 100 μl of PBS suspension using the RNeasy minikit (Qiagen, Hilden, Germany), according to the manufacturer’s protocol. UV inactivation of the sample was done by 10 min radiation under an UV lamp (UVP, UVG-54 254 nm). The virus samples used in this study where the following: HuNoV GI.P7-GI.7 (1.19 x 10^12^ RNA copies/g of stool), HuNoV GII.P16-GII.2 (7.67 x 10^10^ RNA copies/g of stool), HuNoV GII.P16-GII.3 (1.58 x 10^11^ RNA copies/g of stool), HuNoV GII.P4-GII.4 (5.50 x 10^12^ RNA copies/g of stool, MN248513), HuNoV GII.P4-GII.4 (1.01 x 10^12^ RNA copies/g of stool, MN248518), HuNoV GII.P17-GII.6 week 0 (1.12 x 10^13^ RNA copies/g of stool, MN248514), HuNoV GII.P17-GII.6 week 1 (7.33 x 10^10^ RNA copies/g of stool, MN248516), HuNoV GII.P17-GII.6 week 5 (8.48 x 10^9^ RNA copies/g of stool, MN248517), HuNoV GII.P17-GII.6 week 12 (1.47 x 10^11^ RNA copies/g of stool, MN248515), MNV.CW3 (9.3 x 10^6^ TCID_50_/mL) and MNV.CR6 (9.9 x 10^8^ TCID_50_/mL).

### Injection of zebrafish larvae with HuNoV or MNV in the yolk

Three dpf zebrafish larvae were anaesthetized by immersion for 2–3 minutes in Danieau’s solution containing 0.4 mg/mL tricaine (Sigma-Aldrich, Saint Louis, Missouri, stock solution 4 mg/mL in Na_2_HPO_4_, pH 7–7.5). Thereafter, the zebrafish larvae were transferred to a petri dish (92x16mm) with grooves of a mold imprint (6 rows, one side of 90° the other of 45°) in 1.5% agarose. A dissection needle was used to gently orient the zebrafish larvae so that they were lying on their dorsal side with the yolk facing upwards. Injection needles were pulled using glass capillaries (WPI, Sarasota, Florida, TW100F-4) and a Micropipette Puller fitted with a heat filament (Sutter Instruments, Novato, California). In every experiment, the injection needle was calibrated to ensure the precision of the injection volume. Microinjection was done using a M3301R Manual Micromanipulator (WPI) and a Femtojet 4i pressure microinjector (Eppendorf, Hamburg, Germany). Each zebrafish larvae was injected with 3 nL of virus (HuNoV GII.2, HuNoV GII.3, HuNoV GII.4, HuNoV GII.6, HuNoV GI.7, MNV.CW3 or MNV.CR6), while negative control zebrafish were injected with 3 nL of PBS. After injection, zebrafish larvae were transferred to 6-well plates with Danieau’s solution and further maintained in an incubator with a 14/10 h light/dark cycle at 32°C. Every day post injection, the general condition of the zebrafish larvae (e.g. posture, swimming behavior or signs of edema) was observed in order to record clinical signs of virus infection, and 10 zebrafish larvae were collected into 2 mL tubes containing 2.8 mm zirconium oxide beads (Precellys/Bertin Technologies, Montigny-le-Bretonneux, France) and stored at -80°C. To determine the 50% infectious dose (ID_50_), 10-fold dilutions up to 1/10,000 of a HuNoV GII.P7-GII.6 PBS suspension were used to inject zebrafish larvae, as described above. The ID_50_ was defined as the virus inoculum necessary to result in the detection of a significant increase of viral RNA lasting for more than one day pi in 50% of infected zebrafish larvae.

### Antiviral treatment

2′-*C*-methylcytidine (2CMC) was obtained from Carbosynth Limited, Compton, United Kingdom; a stock solution was prepared in DMSO (VWR Chemicals, Radnor, Pennsylvania). Treatment, via immersion, with 4 mM 2CMC started 1 day prior to injection with HuNoV GII.6 or HuNoV GII.4, thus in 2 dpf embryos (10 per condition, manually dechorionated using 2 fine tweezers), and was replenished every 12 h until the end of the experiment. The potential toxicity of 2CMC was evaluated beforehand and a non-toxic concentration was selected to treat injected zebrafish larvae.

### Tissue homogenization and RNA extraction

Zebrafish larvae harvested in Precellys tubes were homogenized with 3 cycles of 5 sec (6300 rpm) with rest intervals of 30 sec (Precellys24, Bertin Technologies). Homogenates were cleared by centrifugation (5 min, 9,000 g) and RNA was extracted using the RNeasy minikit (Qiagen), according to the manufacturer’s protocol.

### RT-qPCR for detection of human and murine norovirus

For detection of HuNoV GII or MNV RNA, a one-step RT-qPCR was performed using the iTaq Universal Probes One-Step Kit (Bio-Rad, Hercules, California), primers and probes used are in S2 Table. Cycling conditions were: reverse transcription at 50°C for 10 min, initial denaturation at 95°C for 3 min, followed by 40 cycles of amplification (95°C for 15 s, 60°C for 30 s) [Roche LightCycler 96, Roche Diagnostics, Risch-Rotkreuz, Zwitserland]. For absolute quantification, standard curves were generated using 10-fold dilutions of template DNA of known concentration.

### Sanger sequencing and genotyping

HuNoV isolated RNA was reverse transcribed by a one-step multiplex RT-PCR using the OneStep RT-PCR Kit (Qiagen), according to the manufacturer’s protocol, primers used are in table S2. The cycling conditions were reverse transcription at 50°C for 30 min, initial denaturation at 95°C for 15 min, followed by 40 cycles of amplification (94°C for 30 s, 55°C for 30 s, 72°C for 60 s) and final extension of 10 min at 72°C. The PCR products were run on a 2% agarose gel. All positive PCR samples were purified using ExoSAP-IT (TermoFisher Scientific, Waltham, Massachusetts) and sequenced with the specific GI and GII primer sets. Viral genotypes were determined with the Norovirus Typing Tool Version 2.0 [39].

### Characterization of the immune response by *ifn*, *mx* and *rsad2/viperin* expression following a HuNoV infection

To generate the cDNA, the ImProm-II Reverse Transcription System (Promega, Madison, Wisconsin) was used. Briefly, a total of 1 μg (ca. 10 μL) of extracted RNA was added to 1 μL of random hexamers and incubated at 70°C for 5 min, followed by 5 min at 4°C. To this reaction mix a total volume of 40 μL containing 8 μl of Improm II 5X reaction buffer, 6 mM MgCl_2_, 0.5 mM deoxynucleoside triphosphate, 40 units of RNase inhibitor, 1 μL of Improm II reverse transcriptase, followed by an incubation at 25°C for 5 min, 37°C for 1 h, and 72°C for 15 min. A qPCR was performed with 4 μL template cDNA using the SsoAdvanced Universal SYBR green supermix, 600 nM of forward and reverse primers for *ifn*, *mx*, *rsad2/viperin* and the housekeeping genes *β-actin* and *ef1a*. Primers sequences were as previously described [40, 41]. Cycling conditions were: polymerase activation at 95°C for 3 min followed by 40 cycles of denaturation at 95°C for 15 s, annealing at 55°C and extension at 72°C for 30 sec (Roche LightCycler 96, Roche Diagnostics). Data was normalized to the housekeeping genes and compared to PBS-injected zebrafish larvae to determine the fold induction of the expression, according to the Livak method [42].

### Sample preparation for viral metagenomics

Human fecal samples and zebrafish larvae were prepared using the NetoVIR protocol, with minor modifications [43]. To preserve the fecal sample for the infection experiments, no virus like particle purification was performed. RNA and DNA were extracted using the QIAamp Viral RNA Mini Kit (Qiagen) according to the manufacturer’s instructions, without addition of carrier RNA. First and second strand synthesis and random PCR amplification for 17 cycles were performed using a modified Whole Transcriptome Amplification 2 (WTA2) Kit procedure (Sigma-Aldrich), allowing for amplification of both RNA and DNA [43]. PCR products were purified with MSB Spin PCRapace spin columns (Stratec, Birkenfeld, Germany) as instructed and library preparation was done using a modified Nextera XT DNA kit (Illumina, San Diego, California) protocol [43]. Libraries were quantified with the KAPA Library Quantification kit (Kapa Biosystems) and DNA size of libraries was obtained using Agilent High Sensitivity DNA Kit on a Bioanalyzer 2100 (Agilent, Santa Clara, California). Sequencing of the samples was performed on a NextSeq500 platform (Illumina) for 300 cycles (150 bp paired ends).

### Genomic analysis

Raw Illumina reads were trimmed for quality and adapters using Trimmomatic (version 0.35), S3 Table. The remaining reads were *de novo* assembled into contigs with SPAdes assembler (version 3.9.0) using the metaspades flag [44]. Contigs were classified using DIAMOND in sensitive mode [45]. A full norovirus genome sequence was obtained for the baseline sample (week 0) which was manually checked by aligning sample reads using BWA-MEM [46]. The assembled human norovirus genome of week 0 was used as reference to align the longitudinal patient samples and the sequence obtained from zebrafish larvae 2 days pi to infer viral changes over time using BWA-MEM [46] and Tablet [47]. Trimmed reads from each sample were mapped to the baseline sample of the patient (HuNoV GII.P7-GII.6 week 0) using BWA 32 to obtain individual sample magnitudes for the analysis of the donor-derived reads. An in-house developed python script (python version 2.7.6) was used to generate a summary of annotated viral genus reads per individual sample. To assess mutations on the viral genome overtime, samples were compared to the sample of week 0 using the package deepSNV from biocLite in R. Sites with less than 10 reads coverage were excluded from the analysis. A site was considered either having a mutation, major or minor variant if respectively ≥ 80%, 50–80% or 10–49% of the reads were different from the reference sequence for a particular nt position. Nucleotide positions with < 100 reads were only included as minor variant if >20% of the reads were mutated compared to the reference.

### Enzyme immunoassay (EIA)

HuNoV structural antigens were detected in infected zebrafish larvae via the RIDASCREEN Norovirus 3rd Generation (R-Biopharm, Darmstadt, Germany), according to the manufacturer’s instructions. Ten zebrafish larvae were harvested 1 h pi or 3 days pi and deyolked. The zebrafish larvae were smashed in 50 μl of ddH_2_O with a pestle in a micro centrifuge tube (VWR, Leuven, Belgium) and debris was removed by centrifugation (10 min, 9,000 g). The supernatant was diluted to a volume of 100 μl. In each EIA run, the positive and negative controls of the kit were included for assay validation and cutoff calculation. The optical density (OD) was measured at 450 nm (Spark, Tecan, Männedorf, Switzerland).

### Western blot

Mock and HuNoV injected zebrafish larvae were deyolked at 3 days pi, then lysed with a pestle in RIPA buffer (Thermo Scientific, Waltham, Massachusetts) in the presence protease inhibitor (Merck KGaA, Darmstadt, Germany) and centrifuged at 9,000 g for 5 min at 4°C. Protein concentration of the lysates were determined using the BCA protein assay kit (Thermo Scientific). BHK cells (purchased from ATCC) transfected with GII.4 construct were used as positive controls. Thirty μg of either mock or HuNoV injected zebrafish larvae lysates were analyzed by SDS-PAGE and western blotting. Baby hamster kidney cells expressing T7 polymerase (BSR-T7 cells, received from Klaus Conzelman, Ludwig-Maximilians-Universitat München, Germany) were used to analyze viral protein expressions of HuNoV GII.4. Briefly, BSR-T7 cells infected with poxviruses expressing T7 RNA polymerase at an MOI (based on the virus titer in chick embryo fibroblasts) of 0.5–1.0 PFU per cell, were subsequently transfected with 1 μg construct containing the full length clone of HuNoV GII.4 using Lipofectamine 2000 according to the manufacturer’s instructions (Invitrogen, Carlsbad, California). To analyze viral protein expression, cells were harvested 24 h post-transfection for western blot analysis. Proteins were separated in 12.5% or 17.5% SDS-PAGE and transferred onto a 0.45 μm nitrocellulose membrane (GVS North America, Sanford, Maine). Membranes were then blocked with 5% milk/PBS-T, washed and incubated overnight with primary antibody against viral proteins NS3 or VPg, which were kindly provided by Professor Ian Goodfellow (University of Cambridge). Membranes were washed extensively, incubated with species-specific secondary antibodies (Li-cor, Lincoln, Nebraska) and viral proteins were detected using Li-cor Odessey CLx imaging system. As housekeeping gene, *β-actin* (Proteintech, 60008-I-Ig, Uden, The Netherlands) was used.

### Inoculation of zebrafish larvae via immersion

Four and five dpf zebrafish larvae were immersed in a 1 mL HuNoV GII.P7-GII.6 suspension diluted in Danieau’s (containing ~ 10^11^ viral RNA copies). After 6 h of exposure to the virus, the larvae were washed with clean Danieau’s, and transferred to a well containing new media and further maintained in an incubator with a 14/10 h light/dark cycle at 32°C. Every day pi, the general condition of the zebrafish larvae (e.g. posture, swimming behavior or signs of edema) was observed in order to record clinical signs of virus infection, and 10 zebrafish larvae were collected into 2 mL tubes containing 2.8 mm zirconium oxide beads (Precellys/Bertin Technologies) and stored at -80°C.

### HuNoV serial passaging in zebrafish larvae

Twenty zebrafish larvae were injected with HuNoV GII.P4-GII.4 as described above and were harvested at the peak of replication (2 or 3 days pi). The zebrafish larvae were smashed in 50 μl of sterile PBS with a pestle in a micro centrifuge tube (VWR, Leuven, Belgium) and debris was removed by centrifugation (3 min, 8,000 g). The supernatant was collected and injected in zebrafish larvae at 3 dpf as described before.

### Immunohistochemistry

HuNoV-infected zebrafish larvae were harvested at 1, 2 or 3 days pi and fixed in 4% formaldehyde overnight at 4°C. The following day the formaldehyde was replaced by 70% ethanol and the zebrafish larvae were embedded in an agarose mold, then processed in paraffin, sectioned and stained as previously described [48]. Immunohistochemistry was performed using antibody TV20 at 1/1000 dilution (kindly provided by Dr. Peter Sander, R-Biopharm) and Anti-VP1 (ab92976, Abcam), 1/500 dilution. Additional staining’s were performed with hematoxylin–eosin (H&E). Microscopy was performed using a Carl *Zeiss Axio Imager Z1* microscope at 10, 40 and 100x magnifications and images were captured and processed with the AxioVision 4.8.2.0 software (Zeiss).

### Statistics

Data was analyzed using GraphPad Prism 7 (Graph-Pad Software) and p values were determined with the nonparametric Mann-Whitney test, where ****p<0.0001, *** p <0.001, ** p <0.01, * p<0.05, and ns is p≥0.05.

## Supporting information

S1 Fig Detection of NS3 in HuNoV GII.4 infected zebrafish by western blot. Western blot analysis of the expression of NS3 in mock (Mo) or HuNoV GII.4-infected zebrafish larvae at 3 days pi. Twenty and 40 μg of the zebrafish larvae lysates were loaded on the gel. M: molecular weight marker.
(TIF)

S2 Fig MNV.CW3 and MNV.CR6 do not replicate in zebrafish larvae. Zebrafish larvae injected with (**A**) MNV.CW3 or (**B**) MNV.CR6 (2–3 independent experiments). Larvae were harvested at different days pi, bars represent the mean values ± SEM of viral RNA levels/zebrafish larva, quantified by RT-qPCR. The dotted line represents the LOD.
(TIF)

S3 Fig Immunohistochemistry and H&E of sagittal sections of infected zebrafish larvae. Immunohistochemistry with VP1-targeting antibodies (**A, C, E**) and respective H&E staining (**B, D, F**) of sagittal sections of HuNoV GII.P7-GII.6-infected larvae harvested at day 3 pi. Images show 5 μm sections at 10x (**A, B**) and 40x magnifications (**C-F**) highlighting the pancreas (arrows) of infected larvae.
(TIF)

S4 Fig Immunohistochemistry detecting HuNoVantigens in infected zebrafish larvae at 1 and 2 days pi. Immunohistochemistry of HuNoV GII.P7-GII.6-infected zebrafish larvae harvested at day 1 pi (**A, C, G, I**) and day 2 pi (**E, K**) [and the respective uninfected controls at day 1 pi (**B, D, H, J**) and day 2 pi (**F, L**)] was performed. Images show 5 μm sagittal sections stained with antibodies targeting VP1 at 10x and 40x magnifications. Viral antigens were detected in the intestine, liver and pancreas (**A, C, E**) as well as in the CHT (**G, I, K**). L: liver, P: pancreas, SB: swim bladder, I: intestine, Y: yolk, C: cloaca, CHT: caudal hematopoietic tissue.
(TIF)

S1 Table Accumulated mutations in HuNoV GII.P7-GII.6 strain over time. HuNoV GII.P7-GII.6 (week 0) was used as the reference sequence, nucleotide changes in this virus over time (week 1, 5 and 12) were detected. A nucleotide change is defined as a mutation, major or minor variant if respectively ≥ 80%, 50–80% or 10–49% of the reads were different from the reference sequence for a particular nt position.
(TIF)

S2 Table Primers and probes. Sequences of primers and probes used [49–52].
(TIF)

S3 Table Sequencing coverage for NGS experiment. (TIF)
