# Supplementary figures and images for: A robust human norovirus replication model in zebrafish larvae
Source: PLoS Pathog. 2019 Sep 19;15(9):e1008009. doi: 10.1371/journal.ppat.1008009 (PMC6752765; doi:10.1371/journal.ppat.1008009)

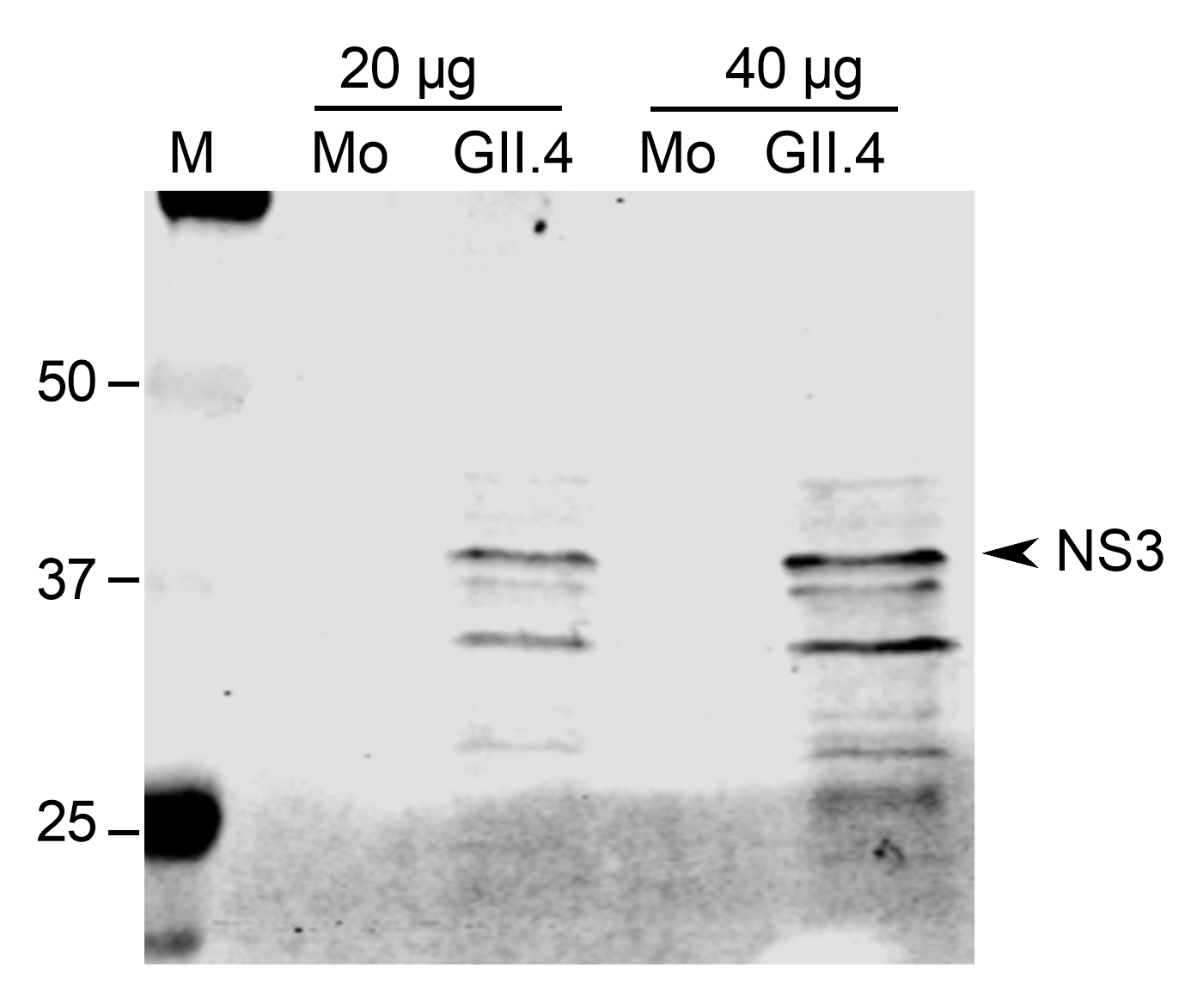

Supplement: S1 Fig — Western blot analysis of the expression of NS3 in mock (Mo) or HuNoV GII.4-infected zebrafish larvae at 3 days pi. Twenty and 40 μg of the zebrafish larvae lysates were loaded on the gel. M: molecular weight marker. (TIF) [file ppat.1008009.s001.tif]

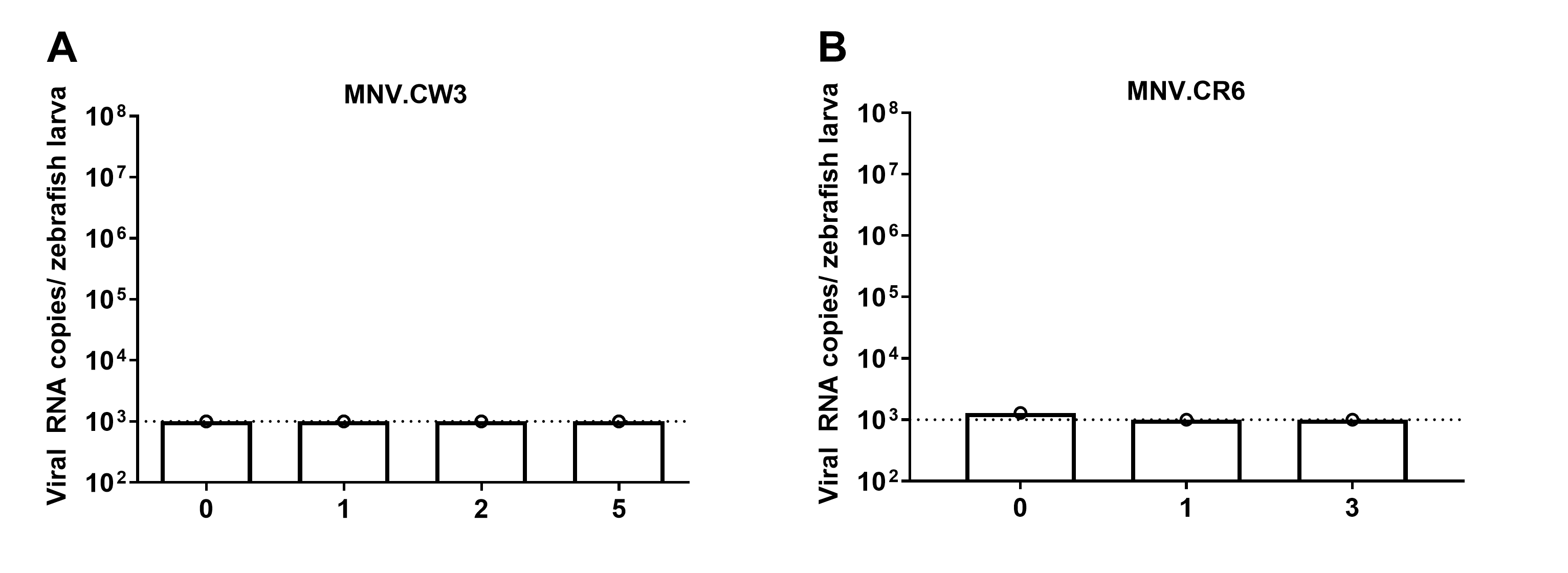

Supplement: S2 Fig — Zebrafish larvae injected with (A) MNV.CW3 or (B) MNV.CR6 (2–3 independent experiments). Larvae were harvested at different days pi, bars represent the mean values ± SEM of viral RNA levels/zebrafish larva, quantified by RT-qPCR. The dotted line represents the LOD. (TIF) [file ppat.1008009.s002.tif]

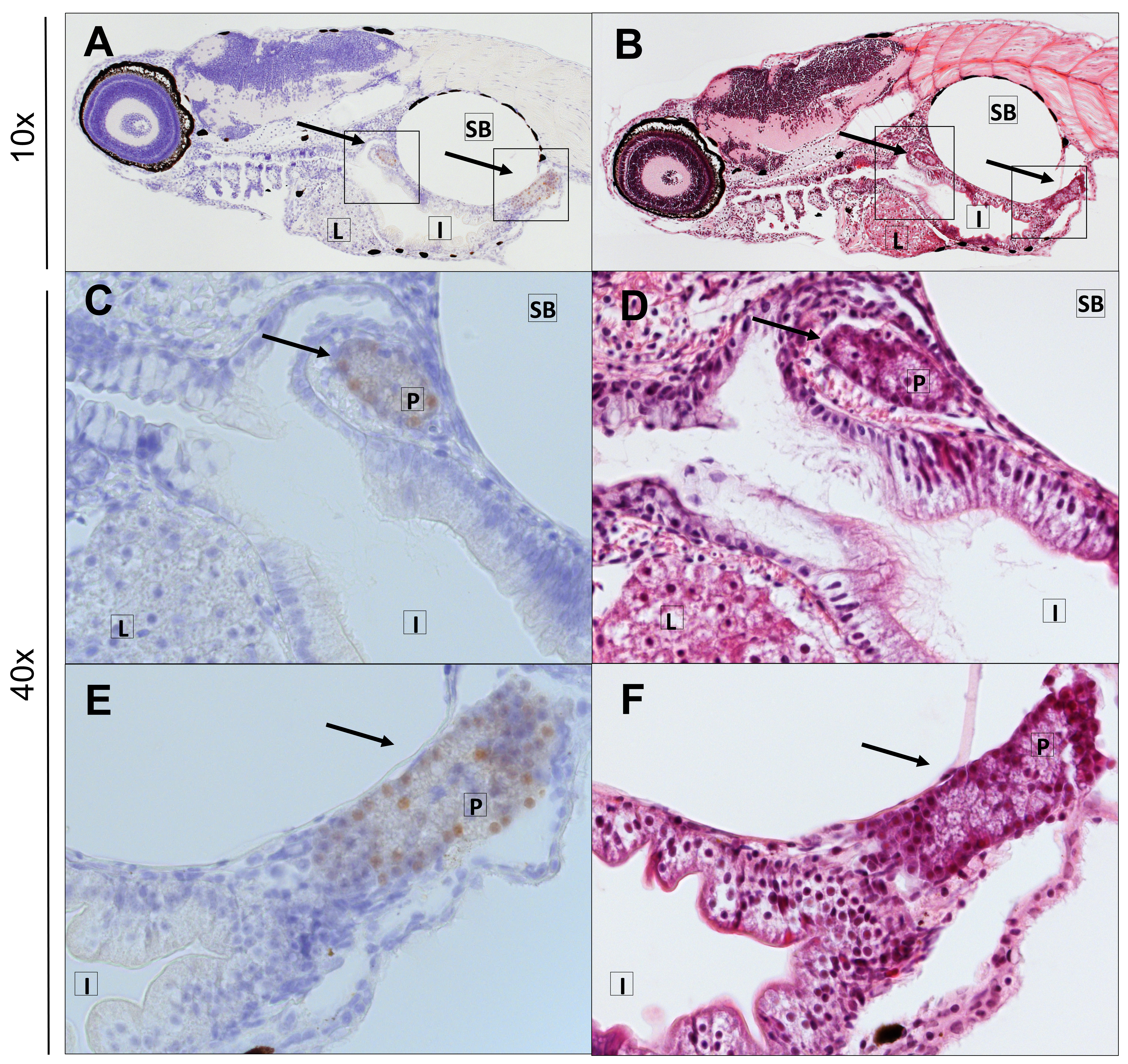

Supplement: S3 Fig — Immunohistochemistry with VP1-targeting antibodies (A, C, E) and respective H&E staining (B, D, F) of sagittal sections of HuNoV GII.P7-GII.6-infected larvae harvested at day 3 pi. Images show 5 μm sections at 10x (A, B) and 40x magnifications (C-F) highlighting the pancreas (arrows) of infected larvae. (TIF) [file ppat.1008009.s003.tif]

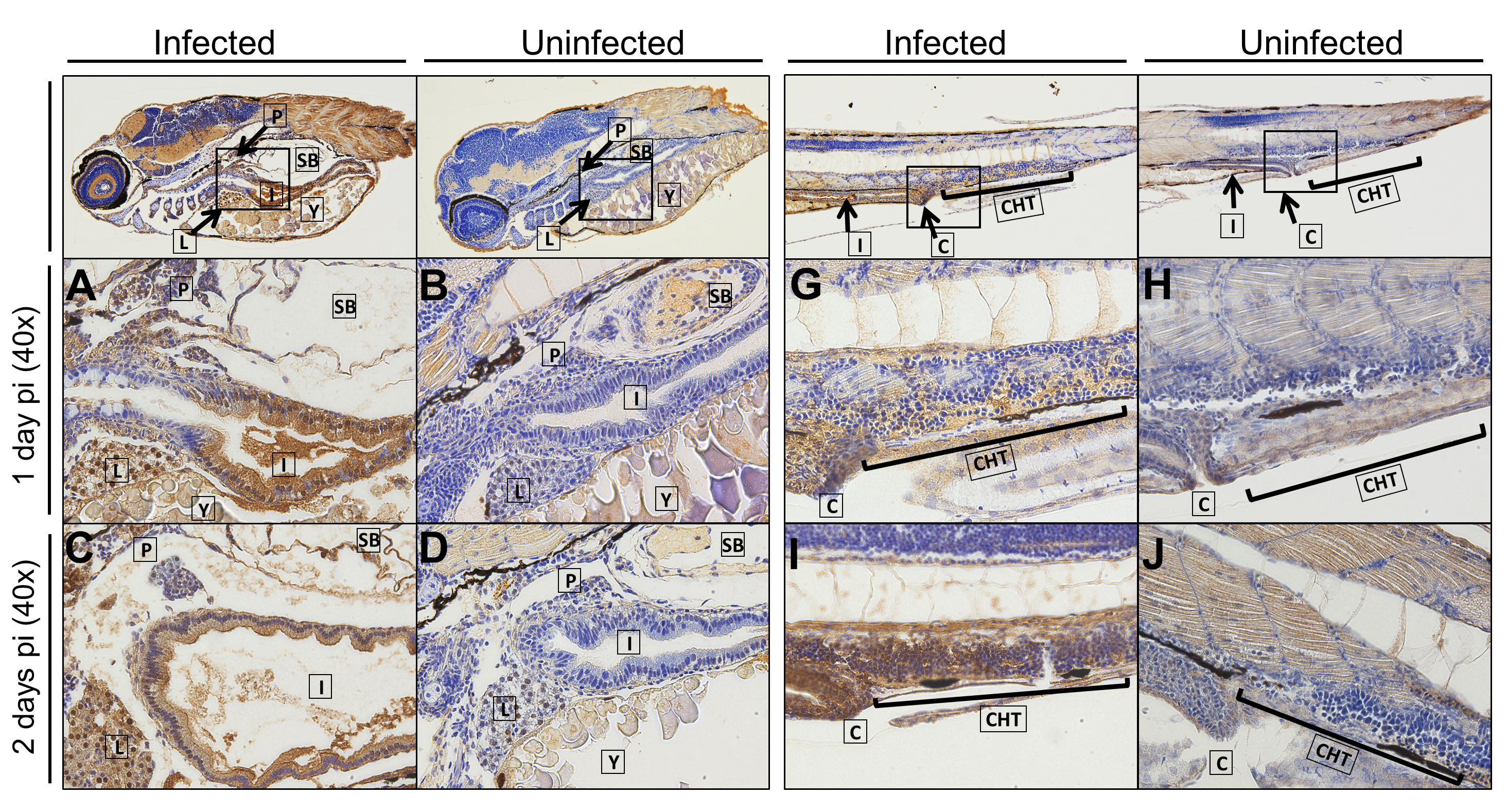

Supplement: S4 Fig — Immunohistochemistry of HuNoV GII.P7-GII.6-infected zebrafish larvae harvested at day 1 pi (A, C, G, I) and day 2 pi (E, K) [and the respective uninfected controls at day 1 pi (B, D, H, J) and day 2 pi (F, L)] was performed. Images show 5 μm sagittal sections stained with antibodies targeting VP1 at 10x and 40x magnifications. Viral antigens were detected in the intestine, liver and pancreas (A, C, E) as well as in the CHT (G, I, K). L: liver, P: pancreas, SB: swim bladder, I: intestine, Y: yolk, C: cloaca, CHT: caudal hematopoietic tissue. (TIF) [file ppat.1008009.s004.tif]

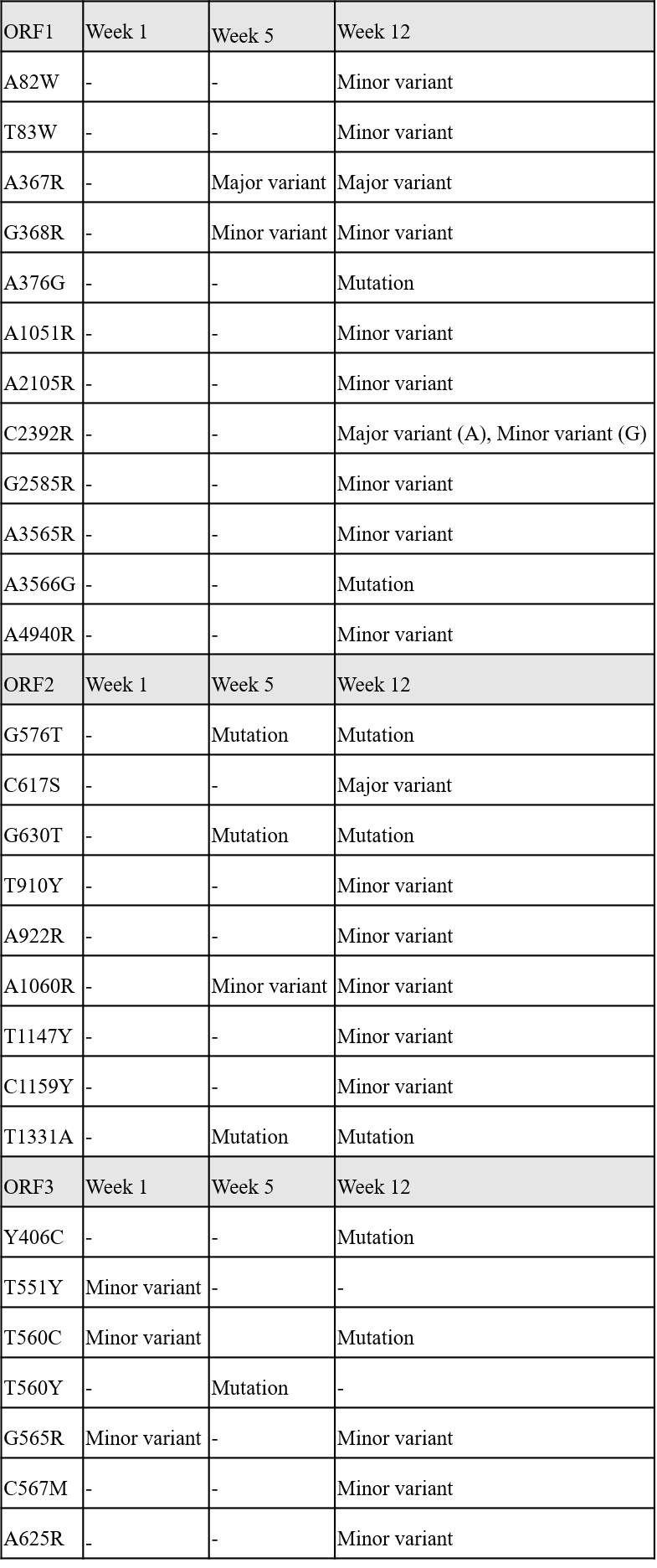

Supplement: S1 Table — HuNoV GII.P7-GII.6 (week 0) was used as the reference sequence, nucleotide changes in this virus over time (week 1, 5 and 12) were detected. A nucleotide change is defined as a mutation, major or minor variant if respectively ≥ 80%, 50–80% or 10–49% of the reads were different from the reference sequence for a particular nt position. (TIF) [file ppat.1008009.s005.tif]

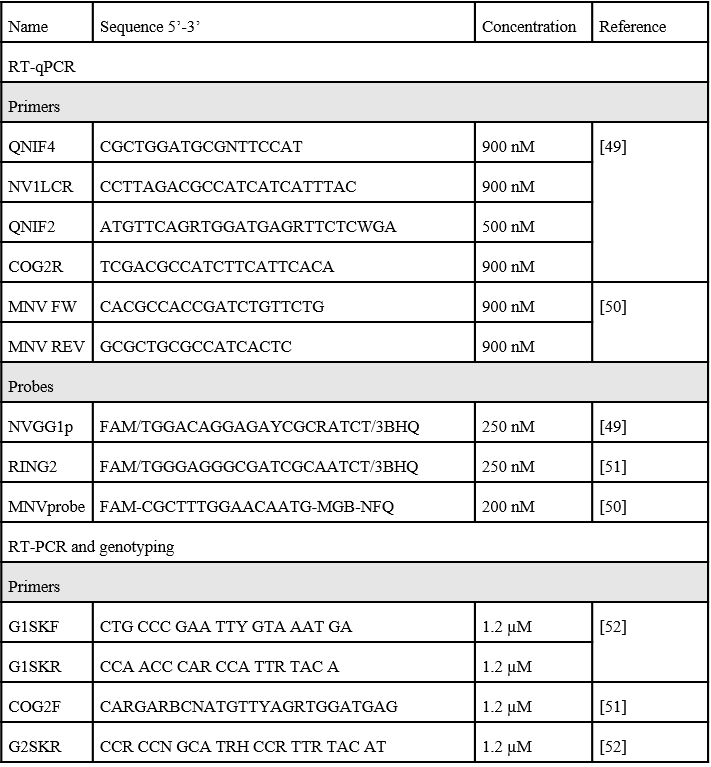

Supplement: S2 Table — Sequences of primers and probes used [49–52]. (TIF) [file ppat.1008009.s006.tif]

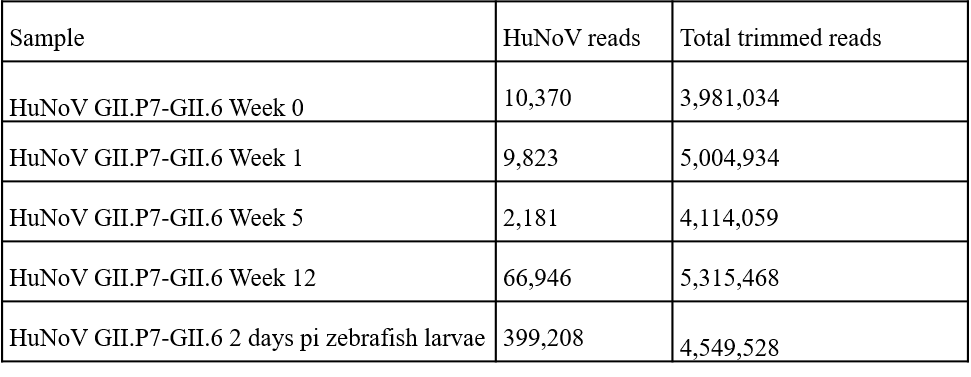

Supplement: S3 Table — (TIF) [file ppat.1008009.s007.tif]
